# Teetotalism and Philanthropy

**Published:** 1899-10-07

**Authors:** 


### TEETOTALISM AND PHILANTHROPY.

If one felt at all inclined to draw morals or cast sneers it would not seem entirely inappropriate to point out how little relation there seems to exist between the loud profession, the wide organisation, and the resulting political importance of the temperance party, and the curiously meagre outcome of all the efforts which have hitherto been made to secure the reformation, or even only the safe custody of those who have become the victims of intemperance. Notwithstanding the enormous prevalence of drunkenness, and the untold misery which it inflicts upon the community, and more especially upon those of the working classes, we find from the annual report of the inspector of the retreats licensed under the Inebriate Acts that there are but 14 such establishments, and that there is an utter absence of any retreat for the accommodation of male patients who are either destitute or are possessed of only strictly limited means. So long as it remains the fact that no provision exists for the reception of poor male inebriates, however anxious they may be to submit themselves to any form of treatment which may be required by their case, and however willing they may be to pay something and to work much for their maintenance, we cannot but feel a lack of sympathy with what is so often by a strange misuse of words spoken of as the temperance movement. Of the enormous evils which result from the immoderate use of alcohol we are convinced; of the desirability of bringing up the young as abstainers from all forms of alcohol we are assured, not only by theoretical considerations, but by practical observation; and of the urgent necessity for a much stricter control over the public sale of the stronger aids to drunkenness than at present exists, everyday experience should teach the necessity; but when we see how little is being done to reform the drunkard by private bodies, and how, even after Acts of Parliament have been passed giving powers for the purpose, these are allowed (as, for example, the Act of 1898) to remain practically dead and inoperative in consequence of the supineness of public bodies, we cannot but feel that either the power and political importance of the temperance party have been exaggerated, or that it is a party with one idea, urgent in forcing this idea on others, but devoid of charity. Probably the latter is the truth. The medical profession has often been accused of lukewarmness in the cause of "temperance." But in truth temperance has but few advocates, and among them are many doctors. The question is one of teetotalism, not of temperance; and perhaps one of the reasons why so many medical men hold aloof from this particular creed is its want of charity. We are so accustomed to commingle curative with preventive treatment, and our work so largely involves rescuing the sinner while we condemn the sin, that our sympathy fails to go out to those who, while making no small parade of their philanthropy, expend their energies on purely preventive measures and leave the victims of alcoholism to simmer in their misery.

